# Dopamine D1 + D3 receptor density may correlate with parkinson disease clinical features

**DOI:** 10.1002/acn3.51274

**Published:** 2020-12-21

**Authors:** Pengfei Yang, William C. Knight, Huifangjie Li, Yingqiu Guo, Joel S. Perlmutter, Tammie L.S. Benzinger, John C. Morris, Jinbin Xu

**Affiliations:** ^1^ Department of Radiology Washington University School of Medicine St. Louis Missouri 63110 USA; ^2^ Department of Neurology Washington University School of Medicine St. Louis Missouri 63110 USA; ^3^ Department of Neuroscience Washington University School of Medicine St. Louis Missouri 63110 USA; ^4^ Department of Physical Therapy Washington University School of Medicine St. Louis Missouri 63110 USA; ^5^ Department of Occupational Therapy Washington University School of Medicine St. Louis Missouri 63110 USA

## Abstract

**Objective:**

Dopamine D2‐like receptors – mainly dopamine D2 receptors (D2R) and dopamine D3 receptors (D3R) – are believed to be greatly involved in the pathology of Parkinson disease (PD) progression. However, these receptors have not been precisely examined in PD patients. Our aim was to quantitatively calculate the exact densities of dopamine D1 receptors (D1R), D2R, and D3R in control, Alzheimer disease (AD), and Lewy body disease (LBD) patients (including PD, Dementia with Lewy bodies, and Parkinson disease dementia); and analyze the relationship between dopamine receptors and clinical PD manifestations.

**Methods:**

We analyzed the densities of D1R, D2R, and D3R in the striatum and substantia nigra (SN) using a novel quantitative autoradiography procedure previously developed by our group. We also examined the expression of D2R and D3R mRNA in the striatum by *in situ* hybridization.

**Results:**

The results showed that although no differences of striatal D1R were found among all groups; D2R was significantly decreased in the striatum of PD patients when compared with control and AD patients. Some clinical manifestations: age of onset, PD stage, dopamine responsiveness, and survival time after onset; showed a better correlation with striatal D1R + D3R densities combined compared to D1R or D3R alone.

**Interpretation:**

There is a possibility that we may infer the results in diagnosis, treatment, and prognosis of PD by detecting D1R + D3R as opposed to using dopamine D1 or D3 receptors alone. This is especially true for elderly patients with low D2R expression as is common in this disease.

## Introduction

Parkinson disease (PD) – one common type of Lewy body disease – is the second most common neurodegenerative disease in people over 60 years old. PD may be difficult to distinguish from other similar conditions, particularly in the early stage of the disease due to overlapping symptom manifestations. Levodopa (L‐Dopa) remains the most effective pharmacologic treatment for PD currently available. However, as the disease progresses, doses of L‐Dopa may cause adverse side effects such as dyskinesia and motor fluctuations. Alternatively, dopamine receptors (DRs) provide another potential target for pharmacotherapy. DRs are divided into 2 main groups: the dopamine D1‐like receptors (D1Rs) and the dopamine D2‐like receptors (D2Rs). Generally speaking, D1Rs (including D1R and D5R) couple to Gαs/olf and induce production of cyclic adenosine monophosphate (cAMP) and the activation of protein kinase A (PKA). D2Rs (including D2R, D3R, and D4R) activate Gαi/o and inhibit cAMP production.^1^ Dopamine (DA) exerts its effect mainly through D1R, D2R, and D3R in the striatum. The quantification of these receptors may help advance early detection and provide a metric of target engagement. Unfortunately, most of the research on this topic uses animal models and only a few studies have unveiled the distribution of D2R and D3R in the human brain via Positron Emission Tomography (PET). Existing PET tracers do not distinguish D2R from D3R because they share 79% sequence homology.^2^ Therefore, in this study, we used a newly developed method previously published by our group to accurately quantify the distribution of these receptors. This method utilizes two tracers in postmortem human brain slices to map the precise D2R and D3R distribution in the striatum and SN of control, Alzheimer’s disease (AD), and PD patients. After the density of DRs was obtained, we correlated the DR densities with clinically observed symptomatic and therapeutic features of these patients.

Cognitive impairment appears in most PD patients categorized as either early‐onset dementia, Dementia with Lewy bodies (DLB), or later onset dementia called Parkinson disease dementia (PDD) – these 3 distinctions can be broadly categorized together as a Lewy body disease (LBD), or PD‐related diseases. After measuring the D1R, D2R, and D3R in postmortem LBD patients, we clarified whether there were differences in DRs among these classifications. We hope that further insights into these distinctions will provide pathological references to aid the diagnosis of PDD and DLB and help in the discovery of new therapeutic targets for these diseases.

## Methods

### Tissue collection

Human brain tissue was collected after death from a total of 21 control, 34 AD, 11 PD, 16 DLB, and 10 PDD cases who were longitudinally assessed by clinical and pathological features. Most control and AD cases were obtained from the Alzheimer Disease Research Center, while most PD‐related cases were obtained from the Movement Disorders Center in the Department of Neurology, School of Medicine, Washington University in St. Louis. Brain removals were conducted after the written consent was obtained from the next‐of‐kin and all procedures were approved by the ADRC ethical committee and in accordance with local ethical committee procedures and best practices.

### Quantitative autoradiograph

Brain tissue sections were made at 20 μm from the striatum and SN separately. After removing endogenous dopamine, sections were incubated with radiotracers [^3^H]SCH23390, [^3^H]Raclopride, and [^3^H]WC‐10 at a concentration of 1.5 nmol/L, 4 nmol/L, and 4 nmol/L, respectively. When treated with [^3^H]SCH23390 and [^3^H]WC‐10, 30 nmol/L of ketanserin and 10 nmol/L of Way‐100635 were used to block the off‐target binding of [^3^H]SCH23390 to 5‐HT_2_ and [^3^H]WC‐10 to 5‐HT_1A_, respectively. Nonspecific binding was determined by applying 1 µmol/L (+)‐Butaclamol, 1 µmol/L S‐(‐)‐eticlopride, and 1 µmol/L S‐(‐)‐eticlopride to block target receptors of D1R, D2R, and D3R, respectively. After incubation, slides were dried, made conductive, and placed into a Beta Imager 2000Z (Biospace, France) to collect autoradiograph images. Images were analyzed using the Beta‐Vision Plus (Biospace, France) software to calculate receptor densities.

### Determination of actual densities of D2 and D3 receptors

It is hard to distinguish D2R and D3R from each other because of the high degree of structural similarity between the two receptors. Many studies have used raclopride as a specific radiotracer for D2R, but this ligand is not able to distinguish between D2R and D3R.^3^ This approach gives a higher reading for D2R density than its actual density. To provide more accurate data, we used a mathematical model previously published by our team^4^ to calculate the actual densities of D2R and D3R via quantitative autoradiography. The equations are shown as follows:

$$\text{D2R}=\frac{b2B1‐b1B2}{a1b2‐a2b1},\;\text{D3R}=\frac{a1B2‐a2B1}{a1b2‐a2b1}$$

where *a*1 and *b*1 are the fractional occupancies of [^3^H]WC‐10 to D2R and D3R. *B*1 is the apparent total receptor density (both DR2 and D3R) measured directly from the autoradiography experiment of [^3^H]WC‐10 in human brain tissues. Likewise, *a*2, *b*2, and *B*2 are the corresponding parameters for [^3^H]Raclopride, respectively. *a*1, *b*1, *a*2, and *b*2 can be readily calculated according to our publication and their values are 0.05, 0.78, 0.71, and 0.19 respectively.

### *In situ* hybridization & Immunohistochemistry

In situ hybridization (ISH) was performed on brain tissue sections to examine the expression of D2R and D3R mRNA according to the manufacturer’s protocol (ACD Inc, CA). Results indicated tiny purple‐reddish granules in the striatum under optical microscopy. After mRNA detection, some slides were blocked with 10% BSA, incubated with the primary antibody (Abcam, MA) for postsynaptic density protein 95 (PSD95), and the corresponding secondary antibody (Sigma, CA). Samples were labeled with a diaminobenzidine (DAB) kit (ThermoFisher, TX), and counterstained with hematoxylin (Sigma, CA), and finally analyzed with Image J software.

### Immunofluorescence (IF)

Brain tissue sections were blocked with 10% BSA, incubated for 10 h with primary antibodies for D2R (Fisher, MA), PSD95, and synaptophysin (Abcam, MA). Samples were then incubated with Alexa 488, 546, or 647‐conjugated donkey secondary antibodies (Life Technologies, CA) to complex with the primary antibodies. Images of the striatum regions were then acquired using fluorescence microscopy (LSM, Zeiss, German).

### Statistical analysis

The receptor densities were calculated as described previously and expressed as fmol/mg tissue. If the data were observed to be lower than 0 (often due to the negligible D2R density), the data were considered to be 0. Data were expressed as mean ± standard deviation (SD). A one‐way analysis of variance (ANOVA) was used to analyze the differences between various disease groups and the Bonferroni method was used for post‐hoc tests. A survival curve was obtained by the Kaplan–Meier method. All the statistical analyses were conducted using IBM SPSS statistics 22.0 software.

## Results

### Physical, neuropathological, symptomatic, and therapeutic features of all cases

All the cases involved a relatively elderly population. The mean age at death showed that individuals in the PD and PDD groups died earlier than the control group (*P* < 0.05). The brain weight of AD patients was significantly lower than that of the control, PD, and PDD groups (*P* < 0.05). This change in brain mass is consistent with the substantial neuronal cell loss and atrophy commonly seen in AD. The neuropathologic evaluation revealed Braak neurofibrillary tangle (NFT) scores of stage V or VI in most of the AD patients, but only mild stages (I to III) in most of the LBD cases. Control cases significantly differed from the other groups on amyloid beta (Aβ) pathology (*P* < 0.01). There were no statistical differences in the age of onset between all groups. It is noteworthy that when discussing the symptomatic and therapeutic features – hallucination, dyskinesia, PD stage, and dopamine responsiveness – between PD, DLB, and PDD groups; we used LBD cases obtained from the Movement Disorders Center as the data received from the AD center had inconsistent evaluation criteria and incomplete data. The results showed that there were no differences between PD, DLB, and PDD groups with the symptomatic and therapeutic features mentioned (Table 1).

**Table 1:** Parts of the characteristics of the groups

	Con	AD	PD	DLB	PDD	*P*
Gender	11 Female/10 Male	20 Female/14 Male	3 Female/8 Male	7 Female/9 Male	2 Female/8 Male	0.145
Age at death(years old)	87.2 ± 9.1	82.1 ± 8.0	76.4 ± 6.8	81.3 ± 6.2	77.4 ± 7.8	*0.002*
PMI(h)	15.4 ± 12.0	13.7 ± 6.3	14.9 ± 10.4	17.2 ± 9.7	11.2 ± 5.6	0.522
Brain weight(g)	1259 ± 174	1134 ± 147	1298 ± 120	1230 ± 152	1325 ± 176	*0.001*
NIA‐Reagan criteria	6 not met	20 high likelihood	3 not met	1 high/ 1 low likelihood/9 not met	1 low likelihood/6 not met	*0.000*
Braak NFT stage	2 stage 0/ 7 stage I/ 8 stage II/3 stage III	2 stage IV/14 stage V/19 stage VI	5 stage I/ 2 stage II/ 4 stage III	9 stage I/ 2 stage II/ 3 stage V /2 stage VI	3 stageI/2 stageII/5 stageIII	*0.000*
Braak AB stage	All normal	All stageC	3 normal/1 stage A/ 2 stageB/5 stageC	1 normal/1 stage A/ 2 stageB/7 stageC	1 normal/2 stageA/ 1 stageB/5 stageC	*0.000*
DLB(McKeith) criteria	‐‐‐‐‐‐‐	‐‐‐‐‐‐	2 no Lewy bodies/1 diffuse type	10 diffuse type	7 diffuse type	*0.000*
Age onset(years old)	‐‐‐‐‐‐	71.3 ± 10.3	63.8 ± 8.4	67.7 ± 9.7	62.9 ± 9.7	0.066
Hallucination	‐‐‐‐‐‐	‐‐‐‐‐‐	3 no/7 yes	2 no/9 yes	0 no/9 yes	0.230
Dyskinesia	‐‐‐‐‐‐	‐‐‐‐‐‐	4 no/6 yes	4 no/7 yes	3 no/ 1 modest/5 yes	0.986
PD stage	‐‐‐‐‐‐	‐‐‐‐‐‐	3 stage2/1 stage 2.5/4 stage3/2 stage4	2 stage2.5/3 stage 3/4 stage4/2 stage5	2 stage2/3 stage 3/3 stage4/1 stage5	0.143
L‐Dopa responsiveness	‐‐‐‐‐‐	‐‐‐‐‐‐	1 modest/9 yes	1 no/1 modest/9 yes	2 modest/7 yes	0.721

Italic for *p*‐value indicates *P* < 0.05. If the total number of different levels of pathological criteria is less than the total number of cases, then some patients have not been diagnosed according to the pathological criteria.

PMI = postmortem interval, NIA‐Reagan criteria were divided into four levels: high, intermediate, low likelihood of dementia, and criteria not met. Braak NFT = Braak neurofibrillary tangle stage, ranging from stage I to VI with increasing histopathologic manifestations. Braak AB stage = Braak amyloid‐beta plaque stage, ranging from stage A to C with increasing histopathologic manifestations. DLB criteria were used to indicate the presence and distribution of Lewy body related pathology, including brainstem predominant type, intermediate or transitional type, diffuse type, or no Lewy bodies. Hallucination and dyskinesia denote there is accompanying hallucination or dyskinesia in PD patients. Dopamine responsiveness denotes whether the patients are responsive to L‐Dopa treatment. No report shown with “‐‐‐‐‐‐”.

### Quantitative calculation of Dopamine D1, D2, and D3 receptors in the striatum and SN of each group

#### Dopamine D1 receptor

**Figure 1:**
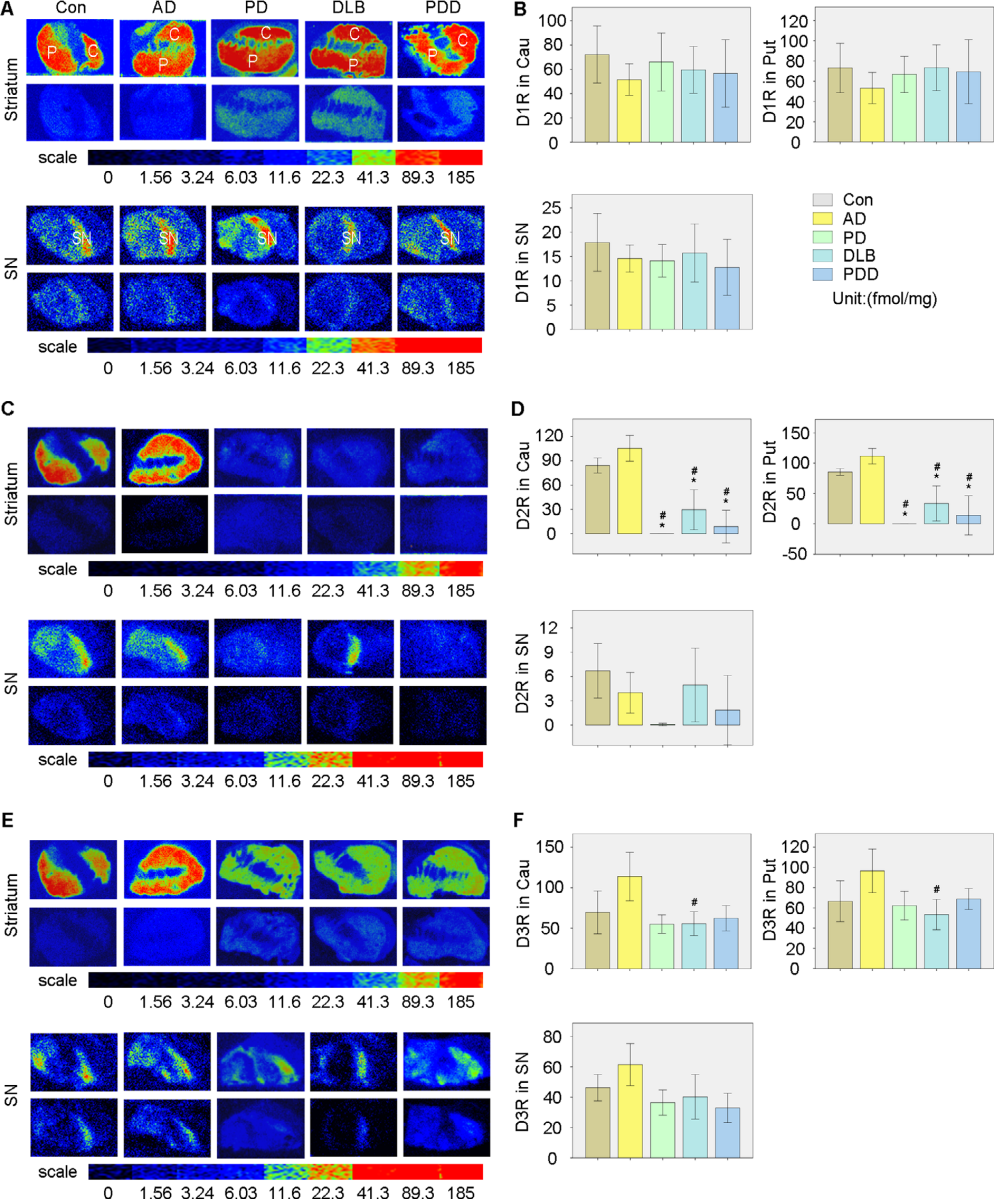
Quantitative autoradiographic analysis of D1R, D2R, and D3R densities in the striatum and SN of all groups. (A) Autoradiograms of specific and nonspecific binding of [^3^H]SCH23390 in the striatum (upper panel) and SN (lower panel) of all groups. (B) Quantitative analysis of D1R densities among all groups. (C) Autoradiograms of specific and nonspecific binding of [^3^H]Raclopride in the striatum (upper panel) and SN (lower panel) of all groups. (D) Quantitative analysis of D2R densities among all groups. (E) Autoradiograms of specific and nonspecific binding of [^3^H]WC‐10 in the striatum (upper panel) and SN (lower panel) of all groups. (F) Quantitative analysis of D3R densities among all groups. *P < 0.05 versus Con, #*P* < 0.05 versus AD.

The calculated data show that there were no statistical differences of D1R in the caudate (Cau) and putamen (Put) between groups. The AD group showed a modestly lower expression when compared with other groups but this result was not statistically significant. D1R density was lower in the SN than in the striatum in all groups, but there were no differences between groups (Fig. 1A and B).

#### Dopamine D2 receptor

The data obtained from the equations revealed that the D2R distribution was quite different from the D1R distribution. D2R expression in the Cau and Put of PD, DLB, and PDD groups was significantly lower than in the control and AD groups (with *P* < 0.05). The PD, DLB, and PDD groups showed no significant differences in the densities of D2R in the Cau and Put. No obvious differences were found in the SN of the different groups (Fig. 1C and D).

### Dopamine D3 receptor

The results showed that the PD, DLB, and PDD groups had lower D3R expression in the Cau and Put than that in the AD group. However only D3R densities in DLB showed statistical differences compared with the AD group (*P* < 0.05). A similar tendency was also found in SN but without statistical significance (Fig. 1E and F). All the data relating to D1R, D2R, and D3R densities in the striatum and SN of each group are shown in Table 2.

**Table 2:** D1R, D2R, and D3R density in striatum and SN in each group

		D1R(fmol/mg)			D2R(fmol/mg)			D3R(fmol/mg)		
		Caudate	Putamen	SN	Caudate	Putamen	SN	Caudate	Putamen	SN
Con	Mean ± SD	72.26 ± 48.77	73.16 ± 50.52	17.90 ± 12.33	84.27 ± 18.67	85.56 ± 11.22	6.71 ± 6.99	69.48 ± 53.31	66.56 ± 40.51	46.21 ± 17.99
AD	Mean ± SD	51.58 ± 35.38	53.19 ± 40.57	14.58 ± 7.74	105.41 ± 42.53	111.60 ± 33.28	4.00 ± 6.84	113.86 ± 80.20	96.59 ± 56.51	61.42 ± 37.85
PD	Mean ± SD	66.09 ± 31.10	66.80 ± 23.30	14.11 ± 5.02	0.00 ± 0.00	0.00 ± 0.00	0.07 ± 0.23	55.00 ± 14.83	62.20 ± 18.33	36.38 ± 11.65
DLB	Mean ± SD	59.56 ± 33.36	73.33 ± 39.10	15.73 ± 10.79	29.66 ± 40.91	33.70 ± 52.00	4.95 ± 7.17	55.62 ± 24.47	53.32 ± 27.07	40.22 ± 23.08
PDD	Mean ± SD	56.74 ± 36.12	69.42 ± 41.29	12.77 ± 8.04	8.79 ± 26.36	14.03 ± 42.09	1.85 ± 5.13	62.26 ± 20.35	68.82 ± 13.47	32.94 ± 11.54

### Analysis of D2R and D3R mRNA expression in the striatum of each group

**Figure 2:**
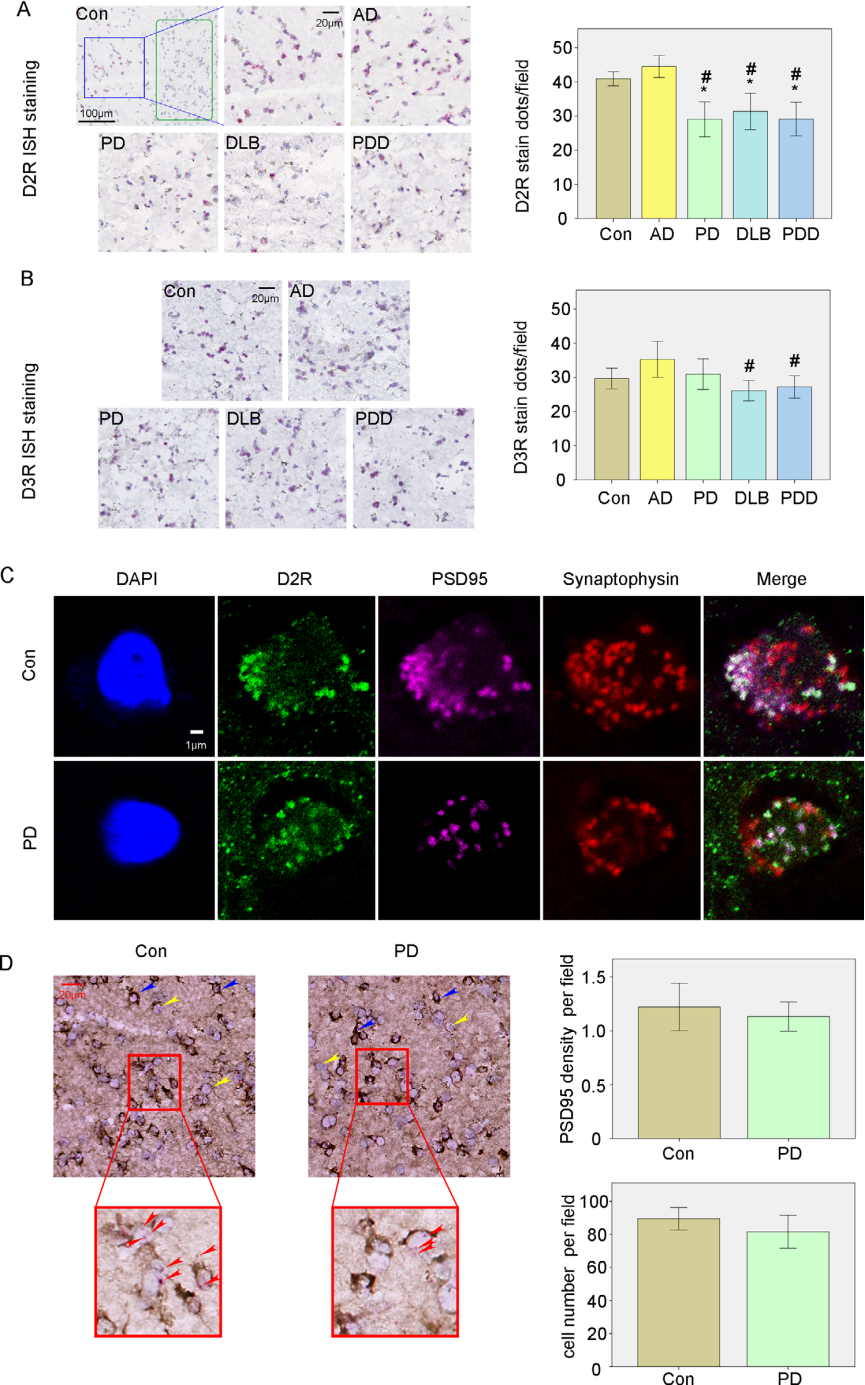
*In situ* hybridization (ISH) analysis of D2R and D3R mRNA and the combination of ISH with immunohistochemistry (IHC) of PSD95 in the striatum of control and PD groups. (A) ISH analysis of D2R mRNA in the striatum of all groups. (B) ISH analysis of D3R mRNA in the striatum of all groups. (C) Localization of D2R expression in the synapse indicated by postsynaptic protein PSD95 and presynaptic protein synaptophysin. (D) Combined ISH with IHC to indicated the expression of D2R mRNA and PSD95. ^*^ *P* < 0.05 versus Con, #*P* < 0.05 versus AD.

The expression of D2R and D3R mRNA were examined using ISH and the results showed that D2R and D3R mRNA only appeared in neurons located in the Cau and Put but not in the internal capsule (green rectangle Fig. 2A Con panel), demonstrating the reliability of our results. Results showed that D2R mRNA expressions in PD, DLB, and PDD groups were significantly lower than in the control and AD groups (with *P* < 0.05, Fig. 2A). D3R mRNA also showed reduced appearance in DLB and PDD groups when compared with the AD group (with *P* < 0.05, Fig. 2B).

### D2R expression showed more colocalization with PSD95 which are not significantly decreased

IF results showed that much of the D2R staining colocalized with PSD 95, a postsynaptic protein, but the colocalization with synaptophysin, a presynaptic protein, was less frequent in the control and PD group (Fig. 2C). Results between PD and control groups show only a minor and not statistically different reduction in PSD95 expression (PSD95 density/per field) in the striatum (Fig. 2D). No differences in cell densities (cell numbers/per field) of the striatum were found between control and PD groups (Fig. 2D).

### Comparing the expression of D1R, D2R, and D3R in different regions of all groups

**Figure 3:**
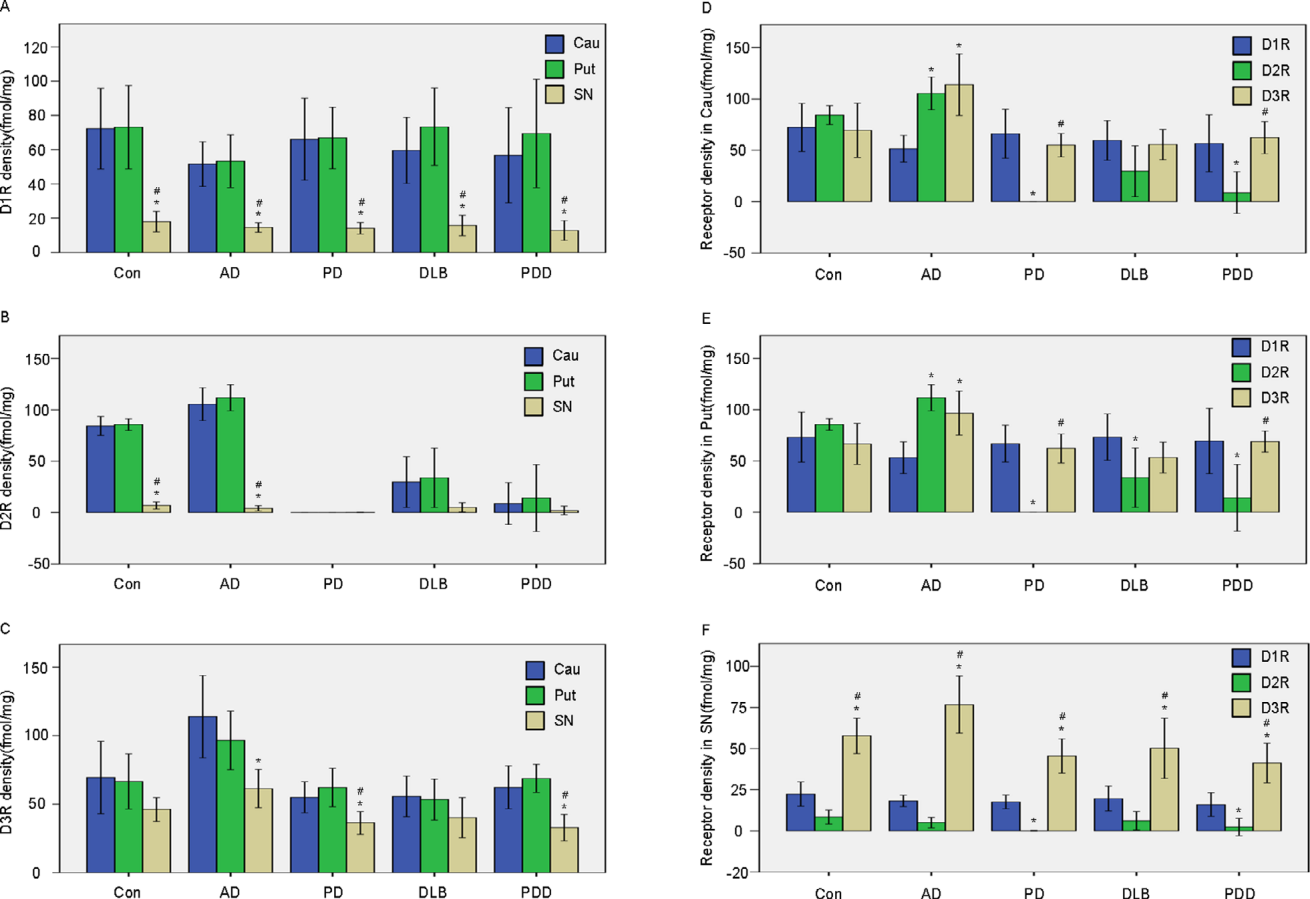
Comparison of the expression of DRs in the different regions of all groups. (A) Quantitative analysis of D1R in the Cau, Put, and SN of all groups. (B) Quantitative analysis of D2R in the Cau, Put, and SN of all groups. (C) Quantitative analysis of D3R in the Cau, Put, and SN of all groups. *p < 0.05 versus Cau, #*p* < 0.05 versus Put. (D) Quantitative analysis of different dopamine receptors in the Cau of all groups. (E) Quantitative analysis of different dopamine receptors in the Put of all groups. (F) Quantitative analysis of different dopamine receptors in the SN of all groups. *P < 0.05 versus D1R, #*P* < 0.05 versus D2R.

We compared the expression of related DRs in different regions in all groups (Figure 3A–C) and found a lower density of D1R in the SN than in the Cau and Put in all 5 groups. D2R expression was greatly reduced in the SN compared to the Cau and Put in control and AD groups. However, these differences were not observed in PD, DLB, and PDD groups. This is mainly due to the remarkable decline of D2R in the Cau and Put in PD, DLB, and PDD patients when compared with control and AD patients. D3R in the SN was also statistically lower than in the Cau and Put in AD, PD, and PDD groups (*P* < 0.05). While similar results were also found in the control and DLB groups but were not statistically significant. It is important to note that DR densities were found to be comparable in the Cau and Put in all these groups, even at the individual patient level (data not shown).

### Comparing the expression of different dopamine receptors in the Cau, Put, and SN region in all groups

We compared the expression of DRs in the related regions of all five groups (Fig. 3D–F). There were no differences between the three receptor densities in the striatum of the control group. D1R was found to be lower than D2R and D3R in the striatum of the AD group. While D2R showed remarkably lower expression in the striatum of the PD, DLB, and PDD groups – these results were found to be statistically significant (*P* < 0.05) when compared with D1R and D3R, with the exception being the Cau of the DLB group. D3R was consistently significantly higher (*P* < 0.05) in SN compared to D1R and D2R in all groups.

### Correlation between DRs densities with PD associated symptomatic and therapeutic features

**Figure 4:**
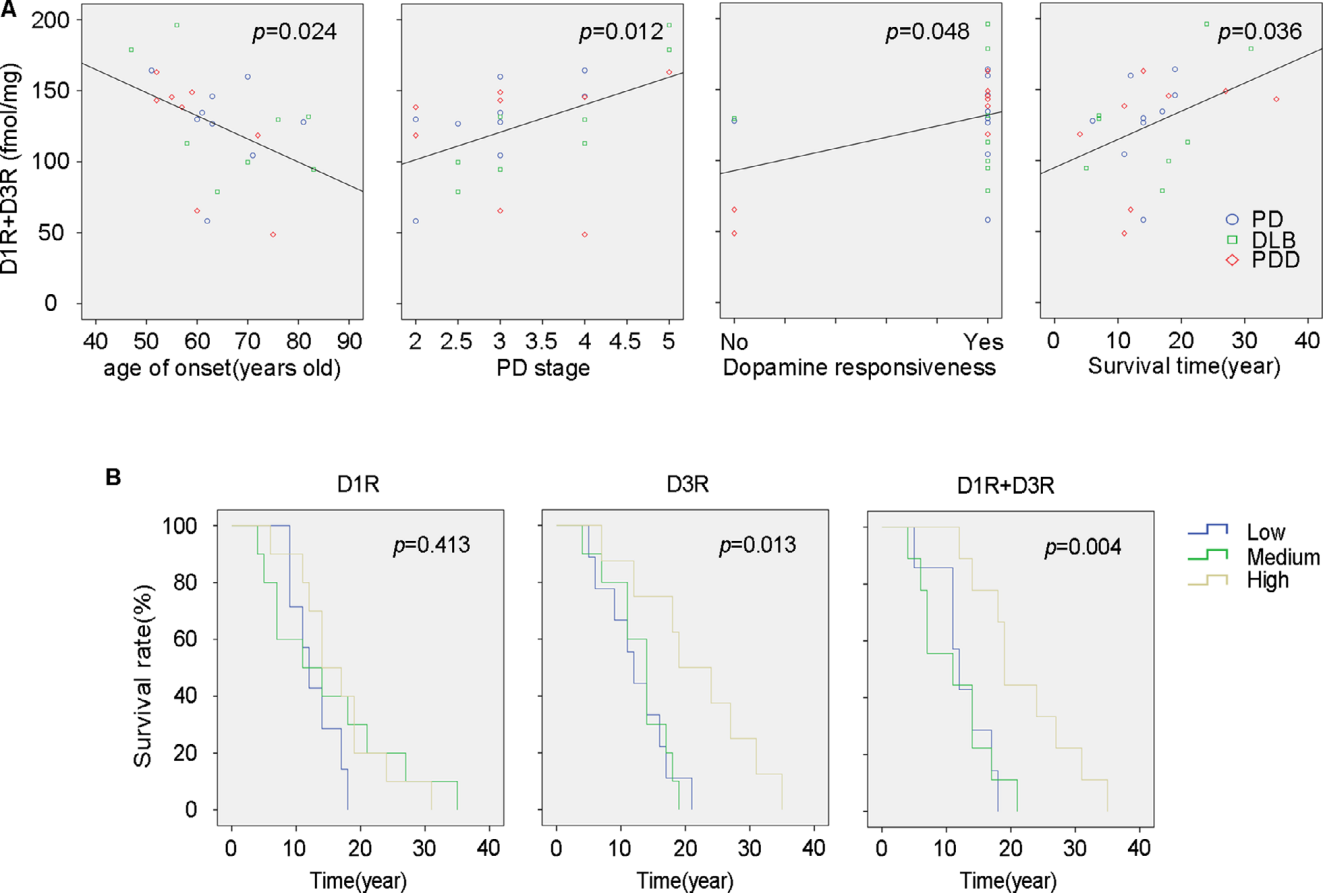
Relationship between D1R + D3R density in the striatum with PD associated symptomatic and therapeutic features. (A) Linear regression analysis between D1R + D3R in the striatum with the age of onset, PD stage, dopamine responsiveness, and survival time are shown with R^2^ values of 0.160, 0.243, 0.203, and 0.178, respectively. (B) Kaplan–Meier survival curves of D1R, D3R, and D1R + D3R made with low, medium, and high‐density levels.

We found slight differences in receptors densities between Cau and Put. These measurements were averaged to reduce noise for a whole striatal measurement. We then evaluated the correlation between striatal and SN DR densities with PD associated clinical manifestations: age of onset, hallucination, dyskinesia, and PD stage. We also correlated DR densities with PD associated therapeutic features: dopamine responsiveness and survival time. The results show that most features have a closer correlation with receptors densities in the striatum than in the SN (Table 3). Striatal D1R density significantly correlated with dopamine responsiveness, whereas striatal D3R density correlated with survival time. The combination of striatal D1R and D3R (D1R + D3R) closely correlated with age of onset, PD stage, dopamine responsiveness, and survival time (Fig. 4A). These findings suggest that when these receptors are analyzed together, D1R + D3R may relate to these symptomatic and therapeutic features more strongly than when analyzed individually.

**Table 3:** Correlation between D1R and D3R with symptomatic and therapeutic features in Striatum of PD‐associated disease

		Striatum			SN		
		D1R	D3R	D1R + D3R	D1R	D3R	D1R + D3R
Age on onset	Coefficient	−0.347	−0.240	−0.450^*^	−0.071	0.464^*^	0.449^*^
	Sig.	0.076	0.229	0.024	0.719	0.026	0.032
Hallucination	Coefficient	0.092	0.049	0.207	−0.211	−0.023	−0.111
	Sig.	0.648	0.809	0.320	0.281	0.916	0.615
dyskinesia	Coefficient	0.196	−0.073	0.211	−0.183	−0.410	−0.392
	Sig.	0.327	0.719	0.311	0.351	0.052	0.064
PD stage	Coefficient	0.362	0.224	0.493^*^	−0.242	−0.069	−0.156
	Sig.	0.064	0.262	0.012	0.216	0.755	0.477
Dopamine responsiveness	Coefficient	0.415^*^	0.134	0.399^*^	0.153	−0.290	−0.203
	Sig.	0.031	0.506	0.048	0.436	0.179	0.353
Survival time	Coefficient	0.327	0.450^*^	0.422^*^	−0.073	−0.266	−0.289
	Sig.	0.096	0.019	0.036	0.713	0.220	0.181

Sig., Significance; Coefficient, Pearson correlation coefficient.

### Impact of D1R and D3R densities on overall survival

We arbitrarily divided the PD patients into three groups according to their receptor densities and performed Kaplan–Meier survival analysis to reveal the impact of receptors densities on the overall survival rate. The results revealed that D3R grade (χ2 = 8.700) and D1R + D3R grade (χ2 = 11.217) in the striatum correlated with the overall survival rate much better than did D1R grade (χ2 = 1.767). The survival rate also showed significant differences in high, medium, and low D1R + D3R densities (*P* < 0.01) (Fig. 4B).

## Discussion

It has been widely documented that the densities of D1R, D2R, and D3R may change in PD patients and that these changes may affect the symptomatic and therapeutic features of treatment. Subsequently, it is of great importance to understand the differences in these receptors between control, AD, and LBD patients. However, the results of early studies on this subject are often inconsistent. There might be several reasons for these inconsistencies. For one, early studies often used different species of animals, but rarely used human specimens. Furthermore, as D2R and D3R share 79% sequence homology, it is hard to distinguish D2R from D3R using conventional methods such as PET due to the lacking of selective D2R or D3R radiotracers. Most studies investigating D2R used raclopride to identify the receptors, but these studies fail to reconcile the fact that raclopride binds to D2R and D3R^3^, which in turn inflates the values for D2R density. In this study, we used the mathematical model previously developed by our group to measure the densities of D2R and D3R in a large cohort of control, AD, PD, DLB, and PDD brains. Our results and observations lead us to some main ideas that we would like to assert.

### Loss of D2R may play an important role in PD pathogenesis

D2R was considered to be greatly involved in PD for a long time. Mice with a D2R deficiency^5^ or treated with antisense oligodeoxynucleotides for D2R^6^ show abnormalities in motor activity analogous to symptoms presented in PD such as reduced locomotion and slow and uncoordinated movements. Further, elevated D2R expression was reported to be neuron protective^7^ and benefit motor performance in MPTP‐induced PD mice^8^, and alleviate symptoms in PD patients.^9^ Several D2R agonists have been used in PD interventions: pramipexole, ropinirole, apomorphine, and rotigotine.^9^

Our results indicated that there is a significant decrease in D2R expression in LBD patients when compared with control and AD patients (about 15.9% and 18.83% of non‐PD subjects expression in the Cau and Put), which we considered an important feature of elder PD patients. The D2R mRNA expression significantly reduced in the striatum of PD patients, but the reductions are lesser than seen in the receptor autoradiography studies. A reason for this discrepancy may be that the protein level is not always proportional to the mRNA expression, since protein translation from mRNA may be attenuated in PD brains,^10^ but this warrants further investigations. The reduction in D2R expression from this study is similar to Farkas’s study where the binding of [^3^H]raclopride, which has 11.25 folds preferential binding to D2R than D3R, in PD postmortem putamen is ~ 8% of the control group (PD 3.73 ± 0.07, control 47.97 ± 10.00 fmol/g).^11^ Our results and data from Farkas showed a much lower expression of D2R in PD than reported by others. The low D2R density may result from the high age of our subjects. Several studies show that D2R expression decreases at high age, after a prolonged course of PD, as the disease progresses, or under chronic exposure to dopamine agonists. Striatal D2R mRNA and protein expressions were about 50% in aged rats (18‐ to 20‐month‐old) in comparison to young adult rats (10‐week‐old).^12^ However, the rats' age in the study was 18 months or equivalent to 45 years of human age, they are much younger than our patient for valid comparisons.^13^ One clinical PET study indicated that D2R binding is significantly reduced in the Cau and Put after 3–5 years of PD progression, estimates of this decline are 0.6% per year in healthy subjects and 1.8 to 3% in PD patients.^14^ One study found that PD patients with more serious symptoms (at H&Y stage III and IV) have significantly lower levels of D2R than those with mild symptoms.^15^ A further report indicates that dopaminergic treatment for PD patients induces D2R reductions of 19.0% and 23.5% in the Cau and Put, respectively, as measured by [^11^C]raclopride.^16^ The longer PD duration, more severe disease stage, and higher age may be the reason why the patients in our study had much lower D2R densities.

IF showed that the D2R expression located primarily in the postsynaptic neurons, and the reduction in D2R may not be related to the post synapse neuronal loss in the striatum of PD patients, as we found that the density of postsynaptic protein PSD95 has only a slight decrease in PD group (without statistical significance). And the striatum is not considered the brain region where neuron loss occurs in PD.^17^ The significant decrease of D2R in the striatum of PD patients supports that aging is a prominent risk factor for PD.^12^ Our findings may also explain why D2R agonists are promising dopaminergic monotherapy in early‐stage PD but not in advanced‐stage PD patients.^18^ Although we have shown that decreased D2R may be an important cause of PD in elder people, the uncertainty of this finding in young cohorts remains to be investigated. The change of PSD95 in PD is still in controversy, both an increase^19^ and a decrease of PSD95 in PD models^20^ have been reported.

### D1R and D3R play important roles in PD treatment, progression, and survival

Although most DR agonists used in PD are designed for D2R activation,^21^ and D2R is thought to be the main dopamine therapy target.^18^ Other receptors such as D1R and D3R may also be involved as DR agonists showed reduced neuroprotective effects throughout treatment but the loss of all protective effects was not seen when D2R was knocked out.^22^ It has long been accepted that decreases in D1R density are directly correlated with PD symptoms^23^ and the increased striatal D1R density is a critical indicator for DR agonists’ clinical sensitivity.^24^ While D3R is reported to be decreased in drug‐naive PD patients^25^ and considered as a potential target for PD intervention.^21^ A D1R preferential agonist, dihydrexidine (with D1R:D2R 10 fold selectivity), was reported to attenuate parkinsonian that can be blunted by a D1R antagonist in a rat PD model.^26^ One double‐blind and randomized study indicated that a selective D1R agonist, ABT431, led to a full antiparkinsonian response in advanced PD patients.^27^ D3R activation is also reported to alleviate striatal DA depletion and behavioral deficits in PD mice, and these effects are D3R dependent as the protective effect disappeared in D3R‐/‐ mice or mice with D3R antagonist pretreatment.^28^, ^29^ D3R preferential agonists are also proven to be effective, and show a dose–dependent response, in alleviating the symptoms of PD advanced patients.^30^, ^31^

The protective effects of D1R or D3R activation on PD symptoms may be independent of D2R or play a cooperative/synergistic effect with D2R. D1R‐/‐ and D2R‐/‐ mice showed comparable reductions in locomotion, while D1R‐/‐D2R+/‐ mice showed more severe impairment in locomotive activity.^32^ Further, microinjections of D1R and D2R agonists cocktails in the striatum of PD mice have produced a better motor stimulating effect than that produced by D2R agonist alone.^33^ These results underline the importance of D1Rs and D2Rs in coordinating movements. Similar results were also observed between D2R and D3R. D2R‐/‐D3R‐/‐ mice showed qualitatively similar but significantly more severe behavioral symptoms than D2R‐/‐ mice.^34^ While the use of the D2R/D3R agonists showed more improvements to PD symptoms in rats with striatal overexpression of both D2R and D3R than the improvement in those with D2R alone.^35^ Together, these studies indicate that D1R and D3R might be potential targets for alleviating PD symptoms in late‐stage PD patients with reduced D2R expression.

### The synergistic effect between D1R and D3R

We conducted correlation analyses between D1R, D2R, D3R, D1R + D2R, D1R + D3R, D2R + D3R, D1R + D2R+D3R, D1R/D3R, and D1R‐D3R in the striatum with symptomatic and therapeutic features in all LBD patients (not all data are shown in Table 3). Only D1R, D3R, and D1R + D3R showed a statistically significant relationship with these features (*P* < 0.05). D1R and D3R each showed a close correlation with one of the six clinical features. D1R + D3R showed a close correlation with four of the six clinical features: age of onset, PD stage, dopamine responsiveness, and survival time. This correlation suggests that D1R + D3R might be a better biomarker for LBD features. The synergistic effect between D1R and D3R has been shown both behaviorally and biochemically in previous reports,^36^ but the question remains, how do D1R and D3R synergize?

(1) Synergism between D1R and D3R may lie in heteromers

**Figure 5:**
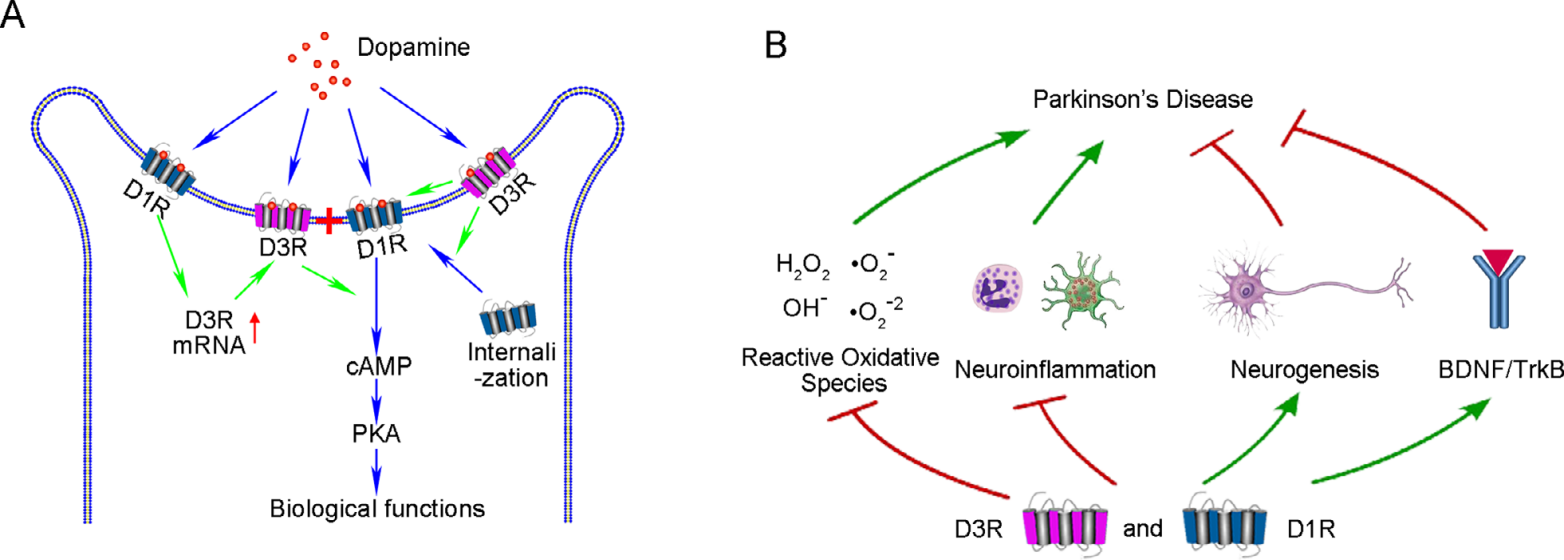
The possible synergistic effect of D1R and D3R in alleviating PD symptoms. (A) Synergism as D1R‐D3R heteromers inside the same neurons to greatly exert the biological functions of DA. (B) Synergism to alleviate pathological injuries or exert protective effects in PD occurrence

D3R shows a great impact on the neuronal regulation of D1R, and *vice versa*. D3R could enhance the density of D1R by down‐regulating the internalization of D1R,^37^ and then forming a D1R‐D3R heteromer, which enhances the affinity of D1R to DA or associated agonists when compared to native state D1R.^38^, ^39^ Moreover D3R helps activate the downstream signal pathway of D1R and increases its sensitivity.^40^ These impacts of D3R on D1R may lead to their synergistic effect. Furthermore, D1R may also enhance D3R expression. The expression of both D3R mRNA and protein in the striatum can be upregulated by repeated D1R stimulation. While this induction can be prevented by SCH23390 (D1R antagonist), but mimicked by SKF39393 (D1R agonist).^41^, ^42^ In this way, D1R promotes D3R expression while D3R results in the oversensitivity of D1R, which may lead to the enhanced physiological effect of both D1R and D3R, and eventually cause that the density of D1R + D3R being more related to the symptomatic and therapeutic features in PD (Fig. 5A).

(2) Synergism between D1R and D3R may lie in the alleviation of pathological injuries or augment of protective effects

Although most of the studies discussing the synergistic effect via the direct formation of D1R‐D3R heteromers, this hypothesis is in debate as D1R and D2R are mostly segregated in the direct and indirect medium spiny neurons (MSNs),^33^ hinting other synergistic effects may exist such as the alleviation of oxidative stress,^43^, ^44^, ^45^ the promotion of Brain‐derived neurotrophic factor (BDNF) expression,^46^, ^47^ the elimination of neuroinflammation,^48^, ^49^ increase of neurogenesis,^50^, ^51^ and enhanced the number of dopaminergic neurons and primary dendrites to exert neurotrophic effects.^52^, ^53^ The proposed synergistic effects of D1R and D3R are summarized in Figure 5B.

Previous reports have suggested that D1R or D3R density might be a predictive indicator for the diagnosis, treatment, or prognosis of PD patients. For example, D1R and D3R expression is proved to be augmented as early as 10–15 weeks before Parkinsonian symptoms in DJ‐1 knockout rat PD models.^54^ Other results obtained from both patients and PD models suggested that a high expression of the D3R gene in the blood is specific for the preclinical staging and therefore be recommended to be used for early diagnosis of PD.^55^ Some reports also suggested that a blockade of dopamine to D1R could decrease L‐Dopa responsiveness^56^ and the insufficiency of D3R closely correlates with damage in respondence to dopaminergic therapy.^57^ In this study, we found the combined density of D1R + D3R in the striatum is more closely correlated to clinical manifestations of LBD patients than D1R or D3R separately.

Another question lies in why D2R agonists help alleviate clinical symptoms but much lower levels of D2R were observed in elderly PD patients in this study. Yet, it should be considered that DR agonists commonly used in clinical applications possess strong D1R and/or D3R activation. Among DR agonists there are two derivatives, ergoline and non‐ergoline. Apomorphine, an ergoline derivative, once considered as D1R/D2R agonist, has been subsequently proved to be a D3R preferring agonist.^58^ Among the non‐ergoline derivatives: pramipexole, ropinirole, and rontigotine are the most widely used dopamine agonists. Pramipexole is considered to be a D1R/D2R/D3R agonist.^59^ Ropinirole and rotigotine, first reported as D2Rs agonists, were also found to bind D3R with higher affinity than D2R.^60^

This is the first report in which D1R + D3R density is more useful in predicting clinical manifestations of LBD patients than D1R or D3R alone. The advantages of our research lie in the fact that we used postmortem brains and a validated method to calculate D2R and D3R. However, the patients selected in our experiment endured a long disease course, had been treated with various drugs for differing periods, and died for distinct reasons. Therefore, our results need to be further validated by clinical trials or animal models.

In conclusion, we observed that D2R densities are significantly decreased in the striatum of later stage LBD patients when compared with control and AD patients. However, there are no significant differences in the density of DRs in the SN among these groups. Ultimately, we believe that the evidence presented has shown that D1R + D3R density may be a more proper influencing factor on the PD features than D1R or D3R alone. Therefore, to design the best individualized intervention, we need to systematically measure the densities of D1R, D2R, and D3R in the striatum.

## Author Contributions

JX contributed to study concept, design, supervision, and obtained funding. PY, WK, HL, and JX contributed to qualitative analysis of data. PY and JX contributed to statistical analysis and interpretation. WK, HL, YG, PY, and JX contributed to preparation of tissue. PY and JX contributed to drafting of the manuscript. PY, WK, and JX contributed to preparation of figures/tables. TSLB, JCM, and JSP contributed to critical revision of the manuscript for important intellectual content.

## Conflicts of Interest

Nothing to report.
